# Loneliness among family medicine providers and its impact on clinical and teaching practice

**DOI:** 10.1038/s41598-025-00688-x

**Published:** 2025-05-08

**Authors:** Frank Müller, Amin K. Charara, Harland T. Holman, Eric D. Achtyes

**Affiliations:** 1 Department of Family Medicine, College of Human Medicine, Michigan State University, Grand Rapids, MI USA; 2 Corewell Health Family Medicine Residency Clinic, Grand Rapids, MI USA; 3 Department of General Practice, University Medical Center Göttingen, Humboldtallee 38, 37073 Göttingen, Germany; 4 Department of Psychiatry, Western Michigan University Homer Stryker M.D. School of Medicine, Kalamazoo, MI USA

**Keywords:** Loneliness and isolation, Health care workforce, Medical education, Health services, Health occupations

## Abstract

Social isolation and loneliness (SIL) are increasingly recognized as health risks. This study examines family medicine providers’ personal experiences with SIL, their perspectives on its importance in clinical practice, and their readiness to incorporate it into medical education. A cross-sectional survey was conducted by the Council of Academic Family Medicine Educational Research Alliance (CERA) among members of major U.S. academic family medicine organizations between October and November 2024. Responses on the 3-item UCLA Loneliness Scale and items assessing attitudes toward SIL in clinical practice and medical education were analyzed using descriptive and bivariate statistics. Among 1,004 respondents (response rate 20.7%), 27.8% had sum scores ≥ 6 on the UCLA-3 item scale indicating considerable loneliness. SIL was particularly prevalent among women (31.1%), underrepresented minorities (36.1%), and Black/African American respondents (40.3%). While 54.1% rated SIL as important in family medicine and 68.2% supported regular screening, only 32.5% agreed that managing SIL falls within clinicians’ responsibility. Providers experiencing SIL themselves reported less frequent patient discussions about loneliness (23.7% vs. 32.0%, *p* = 0.023) and fewer community partnerships. Most respondents (71.0%) reported inadequate resources in their clinics to address SIL. Family medicine educators experience substantial rates of loneliness, particularly among minority groups, at levels exceeding those of their patients and the general population. Personal experiences with SIL appear to influence clinical practices and teaching. Before implementing widespread screening initiatives, the profession must address both providers’ own social connectedness needs and the development of practical clinical resources.

**Supplementary Information:**

The online version contains supplementary material available at 10.1038/s41598-025-00688-x.

## Introduction

Social isolation and loneliness (SIL) have become more prominent in the United States in the wake of the COVID-19 pandemic^1^. Recent research has identified loneliness as a risk factor for developing or worsening of various conditions such as coronary heart disease, obesity, stroke, and dementia^2–4^, though findings have not been consistent among all populations^5^. Social isolation carries an increased premature mortality risk comparable to smoking cigarettes^6,7^ and is linked to increased risks of heart disease and stroke^8^. SIL has worldwide societal and economic consequences beyond individual health. The economic burden and health implications of SIL are undeniable, costing health systems billions annually^9^.

SIL impacts public health in varied ways. In younger population, excessive technology and social media use are linked to fewer face-to-face interactions and feelings of isolation^10^. Meanwhile, older adults with poor social engagement may be at higher risk for dementia^11^, suggesting the need for early screening and intervention. SIL has also been linked to higher rates of depression and anxiety which can worsen physical health outcomes and cause increasing healthcare resource utilization^12^. Further, socially disconnected individuals are less likely to follow medical treatments and preventative care^13^. Managing such risks via targeted strategies including increased SIL assessment integration in healthcare settings may reduce the burden of chronic illnesses and improve public health^14^.

While primary care providers are uniquely positioned to address SIL as the first point of contact for patients^15^, they may also be affected by their own social isolation and loneliness. While research exists on physician burnout, depression, and resilience^16–19^, SIL represents a unique dimension of physician well-being centered on interpersonal connection rather than work-related stressors or mood that requires separate theoretical and empirical consideration. Research shows SIL correlates with these conditions^20,21^ representing a potential pathway for how SIL translates to broader distress—lonely physicians may develop burnout, while burned-out physicians often withdraw from social connections. In recent years, provider well-being has emerged as a prerequisite for delivering high quality patient care, a recognition formalized in the Quadruple Aim framework^22,23^. Impaired provider-wellbeing including frustration, low professional satisfaction, or burnout has been associated with overuse of resources, inappropriate medication prescription and lower patient satisfaction^24–27^.

Despite growing attention to provider well-being, SIL has not been considered in the concept of provider wellbeing and remains understudied. The prevalence of loneliness among family medicine providers is unknown, as is the potential influence of providers’ personal SIL experiences on their clinical approaches to addressing patient loneliness—including their perception of its importance, their available resources, and their views on professional responsibility.

This study aims to understand family medicine physicians personal experiences with SIL, their perspectives on its importance in clinical practice, and their readiness to incorporate it into medical education.

## Methods

This is a cross-sectional online survey among all members of the major U.S. academic family medicine organizations and was conducted between October 15 and November 22, 2024. This study was part of the Council of Academic Family Medicine Educational Research Alliance (CERA) omnibus survey^28,29^. Results are reported following the Consensus-Based Checklist for Reporting of Survey Studies (CROSS)^30^.

### Questionnaire development

The survey consisted of two parts. The first covered demographics of respondents (age, gender, race/ethnicity, self-identification as underrepresented in medicine) and professional characteristics (degree earned, organizational affiliation). To ensure privacy, respondents’ locations were categorized into nine U.S. regions and Canada. The second part of the survey explored personal and professional aspects of SIL through ten items organized in two sections. The first section assessed respondents’ personal experiences using the validated 3-Item UCLA Loneliness Scale^31^, a brief measure used in many studies including large population health studies such as the Health and Retirement Study and English Longitudinal Study of Ageing^32–35^ and has been translated and validated in multiple languages^36–38^. The UCLA 3-item scale was developed as a shortened alternative to the 20-item Revised UCLA Loneliness Scale^39^ and offers advantages over single-item measures by reducing response bias while maintaining sensitivity^34^. The UCLA 3-item scale avoids direct references to “loneliness”, instead measuring the frequency of three experiences using a Likert scale (hardly ever, sometimes, often): lacking companionship, feeling isolated from others, and feeling left out. Responses are summed to create a score ranging from 3 to 9, with scores ≥ 6 commonly indicating significant loneliness^40–43^.

The second section focused on SIL in participants’ work as clinicians and educators and were developed through a structured, iterative process. Initially, we held three team meetings (all authors) to draft preliminary items based on literature on SIL in primary care^14,15,44^. These draft items were then submitted to the CERA committee, where they received feedback from reviewers that led to significant refinements. The CERA steering committee evaluated all items for consistency with the project aims, readability, and evidence of reliability and validity. Subsequently, we worked with a designated CERA Research Mentor to further improve the questions. Finally, the questions underwent pretesting with family medicine educators not included in the sampling frame to assess flow, timing, and comprehensibility before inclusion in the final survey.

Items included question on the perceived importance of SIL in family medicine practice and how often respondents discuss this with their own patients, followed by agreement with statements about clinicians’ role in screening and managing SIL. Final items assessed availability of internal resources and external partnerships to manage SIL in patients as well as teaching activities regarding SIL. The survey questions are available as a supplemental file to this manuscript. All questions were evaluated by the CERA steering committee for consistency and readability, and pretested with external family medicine educators not involved in the study.

### Sample and target population

The target population comprised members of four major academic family medicine organizations: the Society of Teachers of Family Medicine (STFM), North American Primary Care Research Group (NAPCRG), Association of Departments of Family Medicine (ADFM), and Association of Family Medicine Residency Directors (AFMRD). Members represent a diverse group of clinicians, educators, and clinician researchers across academic family medicine departments, residency programs, and primary care research units in the United States and Canada. STFM members are primarily medical educators, NAPCRG members focus on primary care research, ADFM represents academic department faculty, while AFMRD comprises residency program leadership.

From an initial list, program directors, clerkship directors, and department chairs were removed, as they have more administrative roles, resulting in a pool of 5168 members. Of these, 324 were excluded due to undeliverable email addresses (n = 230) or having previously opted out (n = 94), resulting in 4844 eligible participants. After excluding participants that are not involved in regular patient care (e.g. researchers or administrative staff, n = 144) and responses with missing values of key variables (defined as at least two missing values in the UCLA-3 item scale, n = 56), 1004 responses were included in the analyses, resulting in a response rate of 20.7%.

### Survey administration

The survey was conducted between October 15 and November 22, 2024, using SurveyMonkey (Momentive Global Inc., San Mateo, CA). Participants received personalized invitations signed by the presidents of all four sponsoring organizations and up to six reminder emails. Each invitation contained a unique link to prevent multiple submissions from the same participant.

### Statistical analyses

We used descriptive statistics with absolute and relative frequencies, mean and standard deviations (SD) to characterize sociodemographic characteristics of our sample. Missing values were indicated for each variable. Responses of all survey items were displayed as stacked bar graphs.

We then compared survey respondents’ demographics with the general membership data from the sample pool using Chi-Square and Fisher’s Exact or Fisher-Freeman-Halton tests, where appropriate. Information on individuals of the sample pool was limited to 4,736 individuals (97.8% of the eligible participants), as membership data from AFMRD members were unavailable.

We assessed participants experience of SIL using the UCLA 3-Item instrument in two ways: First, we examined the distribution of sum scores (range: 3–9) using Mann–Whitney *U* tests for binary comparisons and Kruskal–Wallis tests for multiple group comparisons. Where Kruskal–Wallis tests indicated significant differences, pairwise post-hoc comparisons were performed with Bonferroni adjustment to control for multiple testing. Second, we investigated the proportion of respondents scoring ≥ 6 (indicating increased SIL, a commonly used threshold^31^) using Chi-square tests or Fisher’s exact/Fisher–Freeman–Halton test, where appropriate. Furthermore, we assessed correlations between Likert-scaled items using Spearman’s rho. For Likert-scaled items outside the UCLA instrument, we examined associations with respondent characteristics using Mann–Whitney *U* tests for binary comparisons and Kruskal–Wallis tests for multiple groups. To test independence between categorical variables, we used Chi-square tests or Fisher’s exact/Fisher–Freeman–Halton tests where appropriate. Responses indicating “I don’t know” or “N/A” were excluded from statistical analyses to allow appropriate assessment of ordinal response patterns. We conducted all analyses using SPSS 29 (IBM, Armonk, NY) and considered *p* < 0.05 as statistically significant.

### Research ethics

This study received approval from the American Academy of Family Physicians Institutional Review Board (Decision#: 19-336). Participants provided informed consent prior to participation. Participation was voluntary and only de-identified data was processed for this survey. All methods were performed in accordance with the relevant guidelines and regulations including Good Clinical Practice and the Declaration of Helsinki.

## Results

**Fig. 1:**
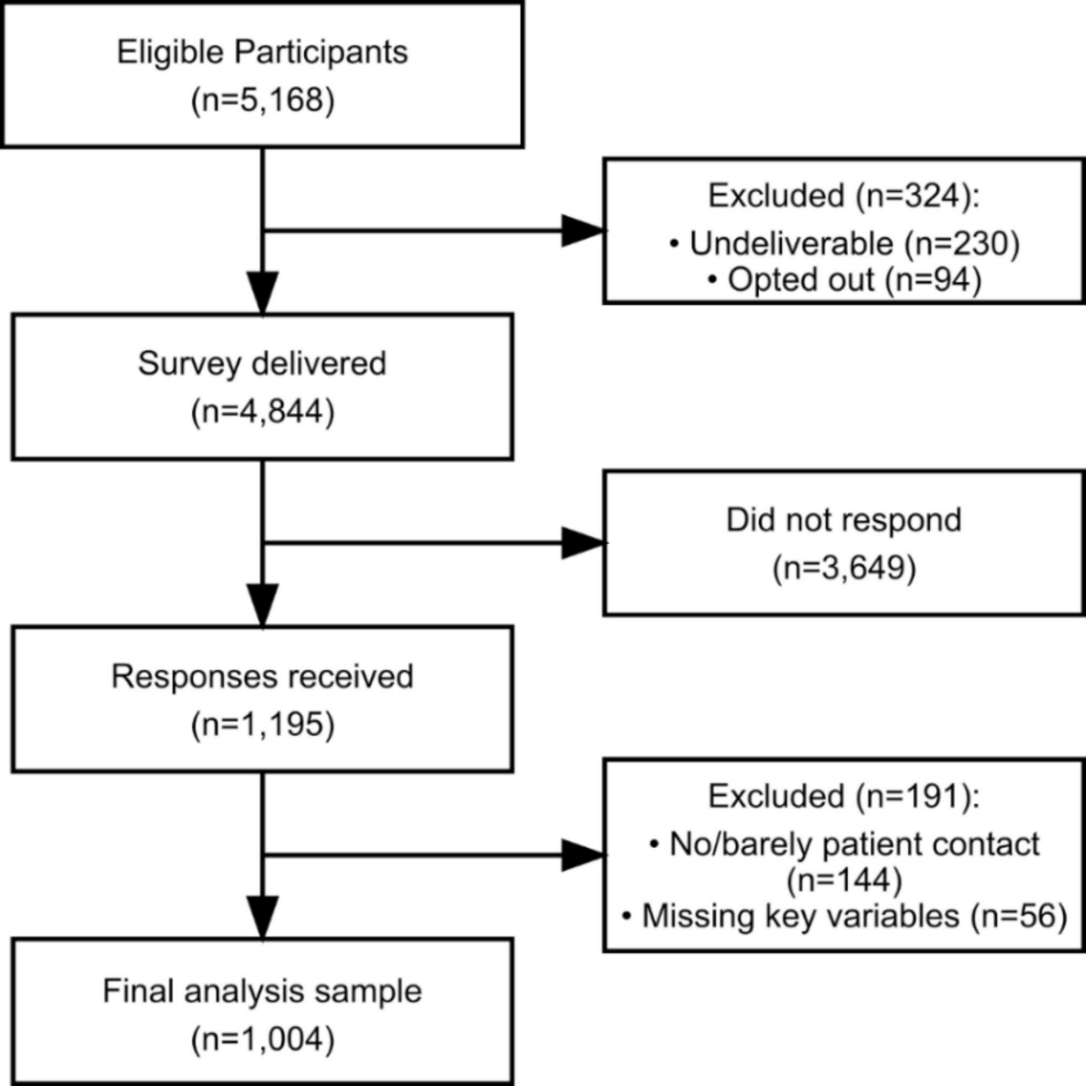
Flowchart of participant inclusion.

Out of 4,844 successively delivered surveys, and after excluding participants who reported not seeing patients regularly and those with missing responses, 1,004 participants were included in the analyses resulting in a response rate of 20.7% (Fig. 1).

### Sample description

The mean age of all respondents was 47.0 years (SD = 11.4). Respondents predominantly worked at allopathic medical schools (55.6%) or non-academic settings (40.3%). Nearly half practiced in urban settings (48.1%), and 64.0% provided care in underserved communities. The majority of respondents were faculty members (49.1%) or associate directors (29.1%), with 58.9% having more than 10 years of practice experience (see Table 1).

**Table 1:** Sociodemographic characteristics of sample pool and included responses of study sample.

		Sample pool (N = 4736*) n (%)	Study sample (N = 1004*) n (%)	*p*
Age	< 30	107 (2.45)	8 (0.94)	0.039
	31–40	1587 (36.35)	312 (36.79)	
	41–50	1208 (27.67)	229 (27.00)	
	51–60	819 (18.76)	179 (21.11)	
	61–70	447 (10.24)	91 (10.73)	
	> 70	198 (4.54)	29 (3.42)	
Gender^†^	Female/Woman	2829 (64.74)	632 (63.58)	0.041
	Male/man	1516 (34.69)	349 (35.11)	
	Genderqueer/non-binary†	25 (0.57)	13 (1.31)	
Race/ethnicity^‡^	American Indian/Alaska Native	24 (0.56)	5 (0.49)	0.467
	Asian	543 (12.58)	120 (11.7)	
	Black/African American	323 (7.48)	64 (6.24)	
	Hispanic/Latino	314 (7.28)	74 (7.21)	
	Middle Eastern/North African	41 (0.95)	16 (1.56)	
	Native Hawaiian/Pacific Islander	14 (0.32)	3 (0.29)	
	White	3057 (70.83)	744 (72.51)	
Region^§^	New England	250 (5.4)	64 (6.4)	< 0.001
	Middle Atlantic	546 (11.79)	128 (12.8)	
	South Atlantic	697 (15.05)	171 (17.1)	
	East South Central	180 (3.89)	39 (3.9)	
	East North Central	817 (17.64)	190 (19)	
	West South Central	353 (7.62)	84 (8.4)	
	West North Central	374 (8.07)	100 (10)	
	Mountain	371 (8.01)	77 (7.7)	
	Pacific	785 (16.95)	128 (12.8)	
	Canada	259 (5.59)	19 (1.9)	
Self-identify as URM	Yes	639 (18.57)	193 (19.3)	0.635
	No	2,802 (81.43)	807 (80.7)	

*Missing data/choose not disclosed in sample pool: Age n = 370, Gender n = 381, Race/Ethnicity n = 651, Region n = 104, URM status n = 1043; in study sample: Age n = 156, Gender n = 3, Race/Ethnicity n = 33, Region n = 4, URM status n = 4. ^†^Genderqueer/Gender non-conforming and Non-binary categories were combined in study sample ^‡^Participants could select multiple options; percentages may sum to > 100% ^§^Regions are grouped as New England (NH, MA, ME, VT, RI, CT); Middle Atlantic (NY, PA, NJ); South Atlantic (PR, FL, GA, SC, NC, VA, DC, WV, DE, MD); East South Central (KY, TN, MS, AL); East North Central (WI, MI, OH, IN, IL); West South Central (OK, AR, LA, TX); West North Central (ND, MN, SD, IA, NE, KS, MO); Mountain (MT, ID, WY, NV, UT, AZ, CO, NM); Pacific (WA, OR, CA, AK, HI).

Compared with the general membership data of the sample pool (n = 4736), the study sample (n = 1004) showed similar demographic distributions. While there were statistically significant differences in age (*p* = 0.039), gender (*p* = 0.041), and geographic region (*p* < 0.001), the absolute differences were small. For example, age groups showed comparable distributions with the largest category being 31–40 years (36.35% vs. 36.79%), followed by 41–50 years (27.67% vs. 27.00%). Gender distribution was also similar, with women representing the majority in both groups (64.74% vs. 63.58%). Race/ethnicity distributions did not differ significantly (*p* = 0.467), with White individuals comprising the largest group in both samples (70.83% vs. 72.51%). The proportion of those self-identifying as underrepresented minorities was also comparable (18.57% vs. 19.3%, *p* = 0.635) (Table 1).

### Provider’s loneliness and social isolation

**Fig. 2:**
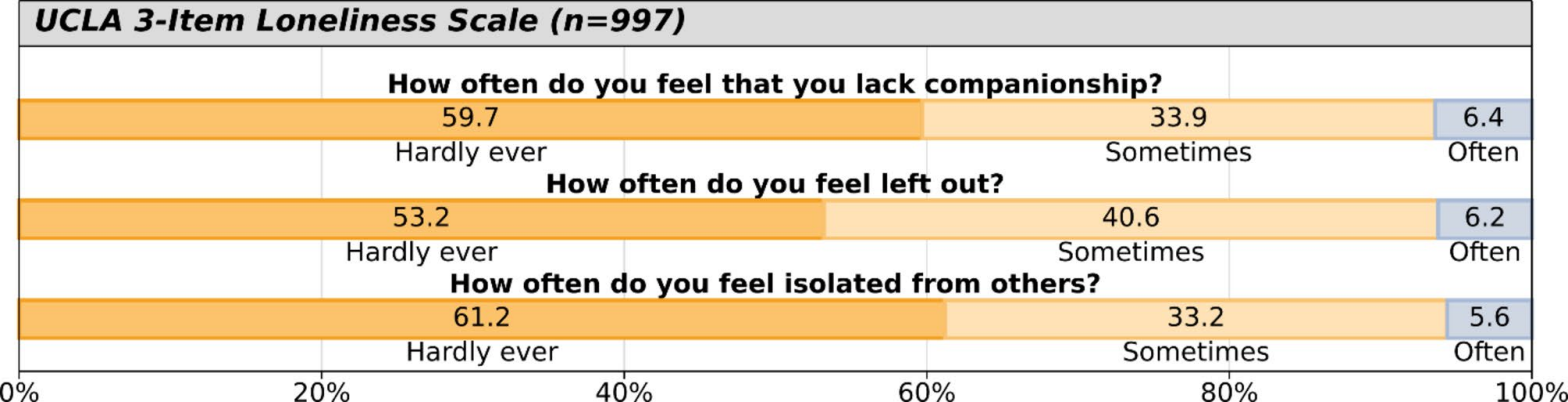
Providers’ self-perceived loneliness and social isolation.

Responses to the UCLA 3-item SIL score are displayed in Fig. 2. Respondents (n = 997) had a mean UCLA sum score of 4.44 (SD = 1.59, range 3–9) and 27.8% of respondents scored ≥ 6, indicating increased SIL.

Women reported higher SIL scores than men (mean score 4.57 vs. 4.21, *p* = 0.001) and had the highest proportion of increased loneliness scores ≥ 6 among all genders (31.1% vs. 22.3% in men and 30.8% in genderqueer/non-binary respondents, *p* = 0.026). Similar results were found among those self-identifying as underrepresented in medicine compared to those who did not (mean scores 4.76 vs. 4.36, *p* = 0.002 or 36.1% scored ≥ 6 vs. 25.7%, *p* = 0.004), and among Black/African American respondents compared to non-Black respondents (mean scores 4.85 vs. 4.41, *p* = 0.036, with 40.3% scored ≥ 6 vs. 27.0% among non-Blacks, *p* = 0.023). Age showed a weak negative correlation with UCLA sum scores (rho = − 0.070, *p* = 0.041), but was not statistically significantly associated with the binary cutoff (*p* = 0.229). No significant differences were found across regions (*p* = 0.557), urban/rural location (*p* = 0.796), or between those serving underserved communities and those who did not (*p* = 0.174).

### Importance of loneliness and isolation and role understanding

**Fig. 3:**
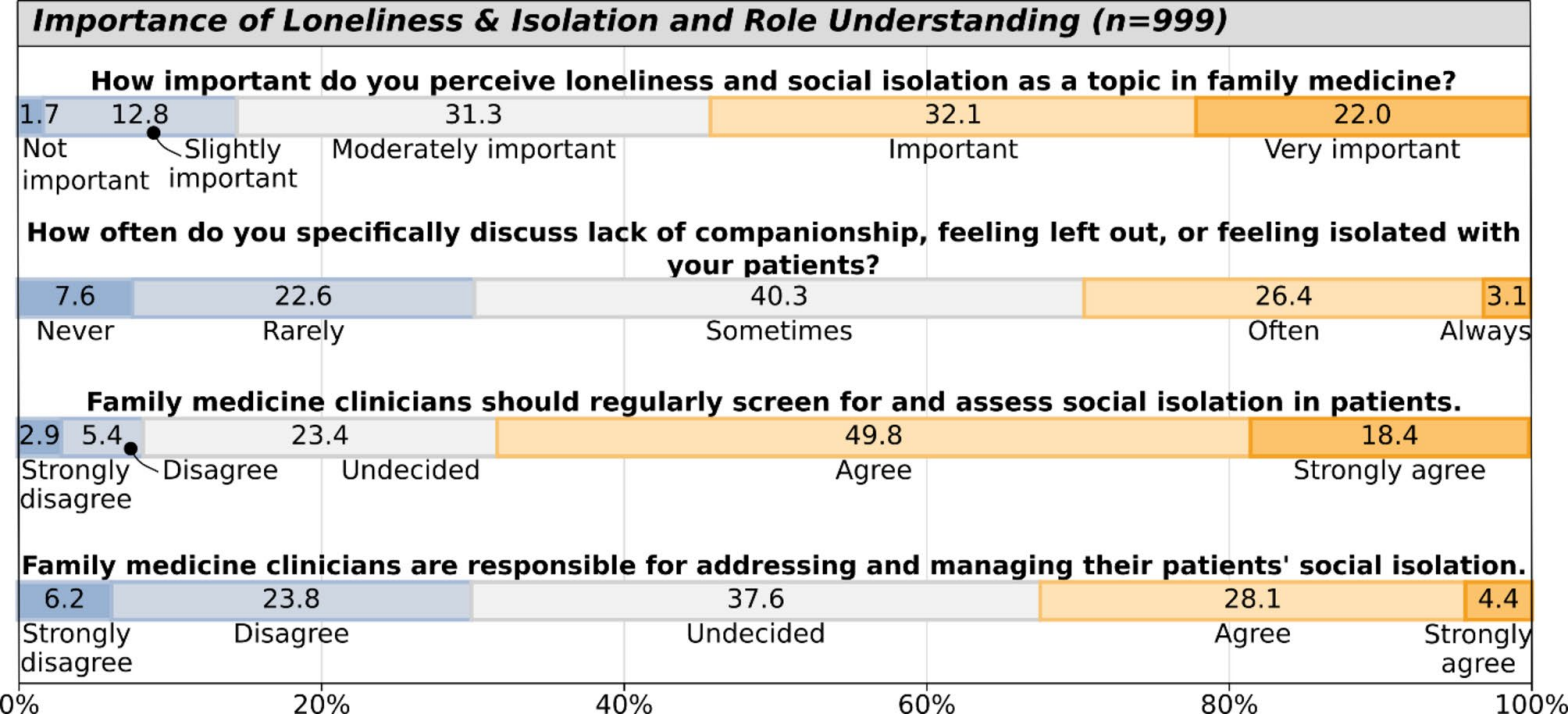
Importance of loneliness.

As shown in Fig. 3, 54.1% of respondents rated SIL as important/very important in family medicine, while 14.5% considered it not or slightly important. Regarding clinical practice, 29.5% reported discussing these issues often/always with patients, compared to 30.2% doing so rarely or never. Most respondents (68.2%) agreed or strongly agreed that family medicine clinicians should regularly screen for SIL. However, only 32.5% agreed or strongly agreed that clinicians are responsible for addressing and managing SIL, while 29.9% disagreed and 37.6% remained undecided.

Several demographic factors were associated with respondents’ views. Women more strongly supported screening for SIL than men (71.0% vs. 63.2% agreed/strongly agreed, *p* = 0.002). Hispanic/Latino respondents rated the importance of SIL higher (64.9% vs. 53.3% in non Hispanic/Latino rated it important/very important, *p* = 0.003) and more strongly supported screening (82.4% vs. 67.1% in non Hispanic/Latino agreed/strongly agreed, *p* < 0.001). Asian respondents showed higher support for clinicians’ management responsibility of SIL (36.7% vs. 31.9% among non-Asians agreed/strongly agreed, *p* = 0.035), while Black/African American respondents more strongly supported screening (75.0% vs. 67.8% agreed/strongly agreed, *p* = 0.015).

Notably, White respondents showed lower levels of support for both screening (65.5% vs. 76.1% agreed/strongly agreed, *p* < 0.001) and management responsibility (30.4% vs. 38.5% agreed/strongly agreed, *p* < 0.001) compared to non-White respondents. Those self-identifying as underrepresented in medicine rated the importance of SIL higher (61.1% vs. 52.4% rated it important/very important, *p* < 0.001) and more strongly supported screening (75.0% vs. 66.7% agreed/strongly agreed, *p* < 0.001).

Respondents who endorsed experiencing SIL themselves (UCLA score ≥ 6) reported less frequent discussions with patients (23.7% vs. 32.0% reporting often/always, *p* = 0.023). No significant associations were found between those experiencing SIL themselves and perceived importance of the topic (57.3% vs. 53.2% rating it important/very important, *p* = 0.566), attitudes toward screening (68.2% vs. 68.2% agreed/strongly agreed, *p* = 0.902), or beliefs about clinicians’ responsibility for management (30.7% vs. 33.3% agreed/strongly agreed, *p* = 0.577).

### Resources to address loneliness and importance in medical education

**Fig. 4:**
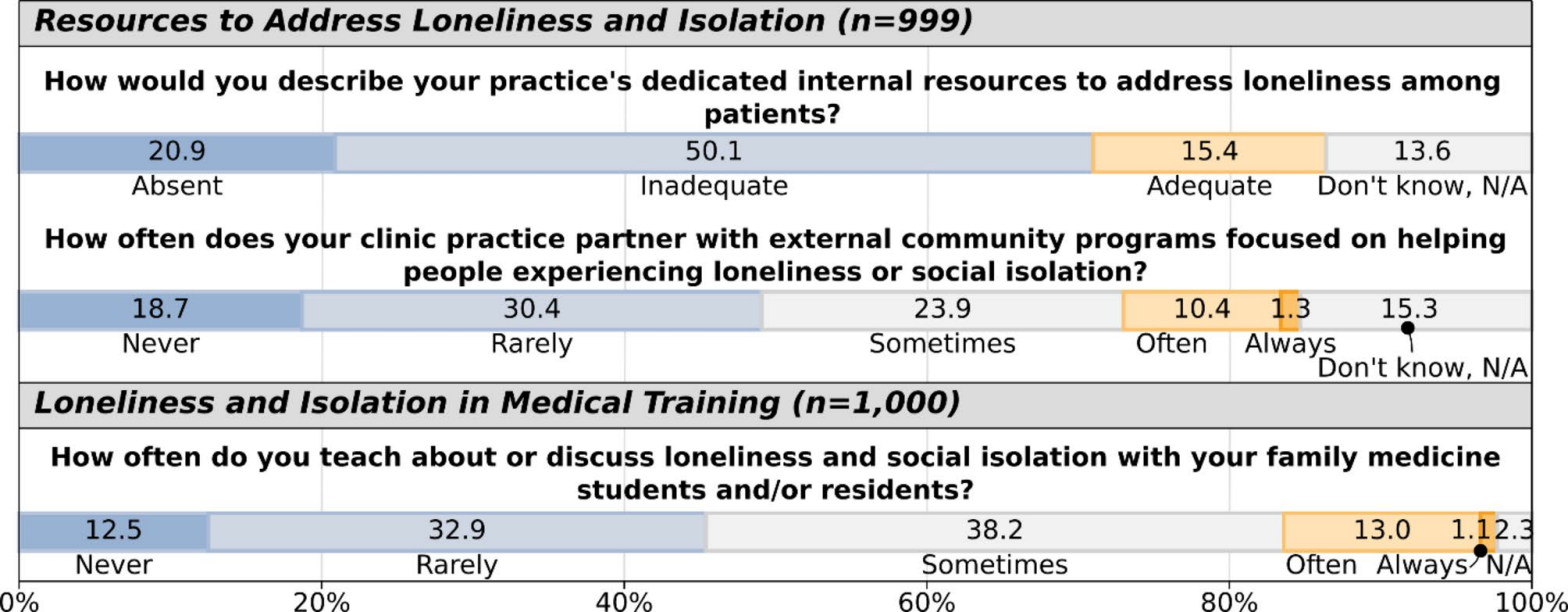
Resources to address loneliness and social isolation.

Internal resources to address SIL were reported as absent or inadequate by 71.0% of respondents (Fig. 4). 49.1% reported never or rarely partnering with community programs. Regarding teaching, 45.4% never or rarely discussed SIL with learners, compared to 14.1% doing so often or always.

Regarding internal resources, women reported adequate resources less frequently than men (12.1% vs. 20.7%, *p* = 0.042). Hispanic/Latino respondents more often reported adequate resources compared to non-Hispanic/Latino respondents (28.8% vs. 14.4%, *p* = 0.006), while Middle Eastern/North African respondents less frequently reported adequate resources (0% vs. 15.7% among non-Middle Eastern/North African descent, *p* = 0.013). Those experiencing SIL (UCLA score ≥ 6) reported adequate resources less frequently than those with lower scores (7.7% vs. 18.2% with UCLA score < 6, *p* < 0.001). For external partnerships with community programs, Asian respondents reported less frequent partnerships (4.2% vs. 12.7% often/always among non-Asian respondents, *p* = 0.020), as did those self-identifying as underrepresented in medicine (9.9% vs. 12.2% often/always among non-URM, *p* = 0.045). Those experiencing SIL themselves also indicated less frequent partnerships (5.5% vs. 14.2% often/always in non-lonely, *p* < 0.001).

Teaching patterns differed only by personal experience of SIL, with those reporting SIL more frequently indicating never/rarely teaching about these topics compared to those with lower scores (50.9% vs. 43.3%, *p* = 0.023).

## Discussion

In this survey of U.S. family medicine provider and educators, we found that personal experience with SIL was common, with 28% of respondents reporting SIL scores that indicate considerable loneliness. This prevalence was particularly pronounced among women, those self-identifying as underrepresented in medicine, and notably Black/African American respondents, where 40.3% reported significant SIL scores.

This prevalence is particularly striking when compared to the rates seen in the very patients these providers treat, where only about 20% report experiencing significant loneliness^44^. The disparity becomes even more apparent when considering population-based studies in Western countries using the same instrument, which consistently show lower SIL prevalence (< 20%)^5,45,46^. Even a recent U.S. study showing SIL rates of 22.8% (UCLA-3 item scales scores ≥ 6^47^) falls below the levels we observed among healthcare providers.

The elevated prevalence appears paradoxical considering the nature of family medicine practice: Clinicians typically spend their days immersed in human connection, both with patients and with allied healthcare team members. This disconnect—experiencing loneliness despite constant human interaction—may be particularly characteristic of “professional helpers”^48–50^. Our findings suggest that being surrounded by people does not necessarily translate into meaningful connection, even for those whose profession centers on human interaction. Considering our sample of educators in family medicine, this social embeddedness can be regarded as even more pronounced, as these providers also engage in teaching residents and students, participate in professional societies, and are more likely to work within larger institutional networks. On the other hand, studies revealed that administrative tasks, increasingly fragment provider-patient interaction, with primary care physicians dedicating 25–50% of patient encounters to computer documentation. Spending more time on paperwork than on direct patient care has led to dissatisfaction and burnout among physicians^51,52^. This reduced quality of interpersonal engagement could contribute to feelings of professional isolation and loneliness in the workplace.

Beyond documentation tasks, limited time for collegial interaction during tightly scheduled days, physical separation in individual exam rooms, and workflow designs prioritizing efficiency over connection all foster professional disconnection. Even in group practices, increasing patient-to-provider ratios and siloed care delivery often impede meaningful engagement with colleagues, creating a paradoxical isolation despite physical proximity to others throughout the workday^22,53,54^.

Given that our sample represents educators who benefit from additional social connections through teaching activities, it is worrisome that SIL might be even more prevalent among primary care physicians in non-academic settings, contributing to physician burnout^55^.

However, these findings warrant careful interpretation within a broader context of SIL research. While humans are inherently social beings, individual differences in personality and temperament mean that optimal levels of social connection vary considerably. This nuance is reflected in previous research showing that large parts of individuals meeting common criteria for SIL do not perceive it as problematic^56^. Such variation in the subjective experience of SIL might help explain recent findings challenging assumed direct relationships between loneliness and health outcomes. For instance, Das et al.^5^ found no significant associations between loneliness and cardiometabolic outcomes in two large longitudinal cohorts of older adults, despite strong theoretical arguments for such connections.

While most respondents in our study considered loneliness and social isolation important in family medicine and supported regular screening, fewer agreed that managing social isolation falls within clinicians’ responsibility. This hesitation about professional responsibility aligns with broader societal views—a U.S. survey found that Americans are nearly evenly split on whether SIL represents a public health issue (47%) versus an individual problem that people need to address themselves (45%)^56^.

Notably, providers experiencing loneliness themselves were less likely to discuss SIL with patients, engage in community partnerships, or teach about the topic, while perceiving fewer practice resources. Future qualitative studies are needed to develop a deeper understanding of the underlying mechanisms of how providers’ personal experiences with loneliness impact their professional engagement with the topic.

Black/African American providers reported the highest prevalence of SIL (40.3%). Black/African American providers often experience racism and discrimination at their workplace e.g. through microaggressions from both patients and colleagues which can contribute to feelings of isolation^57^. This sense of disconnection may be further influenced by the persistent underrepresentation of ethnic minorities in the healthcare workforce^58^. While similar patterns of increased SIL have been observed in the general Black/African American population^59^, our findings reveal a notably more pronounced disparity in the academic family medicine setting, though our cross-sectional data cannot establish causal mechanisms for this observation.

A last finding was that a majority of family medicine providers did not feel adequately equipped to address SIL in their patients despite resources to address SIL in specific patient populations being available in many communities (e.g. Program of All-Inclusive Care for the Elderly (PACE), meals on wheels, or community centers/daycare programs for older adults, or psychosocial clubhouse programs for those with serious mental illnesses)^60^. A review of 22 interventions targeting social isolation and loneliness showed that effective approaches predominantly featured group-based, in-person formats lasting 2–6 months, incorporated active participation through structured learning mechanisms (particularly cognitive behavioral therapy-based), emphasized between-session interactions and community integration, and focused on building sustainable social connections^61^. To effectively address SIL in primary care, future efforts should focus on developing practical clinical tools that bridge the gap between evidence-based interventions and brief interventions or guides for incorporating SIL into motivational interviewing.

### Strengths and limitations

Our study has several strengths and limitations. While the response rate of 20.7% is moderate (but in line with response rates of previous waves of the omnibus survey), our sample closely matched the demographic characteristics of the broader membership pool of the respective professional organizations. Despite demographic similarity between our sample and membership pool, non-response bias remains possible. Respondents may differ from non-respondents in unmeasured characteristics related to SIL. In addition, our findings may not generalize to family medicine providers in non-academic settings. Furthermore, our reliance on self-reported measures of SIL, while using validated instruments, introduces potential reporting bias.

### Outlook

Moving forward, the development of resources tailored to, and connected with, local communities based on evidence-based knowledge is essential for addressing SIL in primary care. However, before implementing widespread screening initiatives, family medicine must address two fundamental challenges. Understanding how providers can maintain meaningful social connections within their demanding professional roles represents the first priority. Creating a more socially connected culture within family medicine itself emerges as the second key challenge. The effectiveness of any patient-directed SIL interventions may ultimately depend on first addressing these professional and cultural dimensions within the discipline.

## Electronic supplementary material

Below is the link to the electronic supplementary material.

Supplementary Material 1

## Data Availability

The data for this study is available at the CERA website: https://www.stfm.org/Research/CERA.
