# Supplementary material for: Loneliness among family medicine providers and its impact on clinical and teaching practice
Source: Sci Rep. 2025 May 8;15:15988. doi: 10.1038/s41598-025-00688-x (PMC12062314; doi:10.1038/s41598-025-00688-x)
Supplement: Supplementary file 1 — Supplementary Material 1 [file 41598_2025_688_MOESM1_ESM.pdf]

SUPPLEMENTARY FILE 1 : SURVEY QUESTIONNAIRE

2024 CERA General Membership Survey

3. Is Social Isolation and Loneliness Considered an Important Topic?

First, in general, how do you feel about different aspects of your own life:

1. How often do you feel that you lack companionship?
   - ◯ Hardly ever
   - ◯ Sometimes
   - ◯ Often

2. How often do you feel left out?
   - ◯ Hardly ever
   - ◯ Sometimes
   - ◯ Often

3. How often do you feel isolated from others?
   - ◯ Hardly ever
   - ◯ Sometimes
   - ◯ Often

Second, we want to understand how these topics impact your work and role as a clinician and/or educator:

4. How important do you perceive loneliness and social isolation as a topic in family medicine?
   - ◯ Not important
   - ◯ Slightly important
   - ◯ Moderately important
   - ◯ Important
   - ◯ Very important

5. How often do you specifically discuss lack of companionship, feeling left out, or feeling isolated with your patients?

- ◯ Never
- ◯ Rarely
- ◯ Sometimes
- ◯ Often
- ◯ Always
- ◯ Does not apply – I am not directly seeing patients

6. How much do you agree with this statement?

Family medicine clinicians should regularly *screen for and assess* social isolation in patients.

- ◯ Strongly disagree
- ◯ Disagree
- ◯ Undecided
- ◯ Agree
- ◯ Strongly agree

7. How much do you agree with this statement?

Family medicine clinicians are responsible for *addressing and managing* their patients' social isolation.

- ◯ Strongly disagree
- ◯ Disagree
- ◯ Undecided
- ◯ Agree
- ◯ Strongly agree

8. How would you describe your practice's dedicated internal resources to address loneliness among patients?

- ◯ Absent
- ◯ Inadequate
- ◯ Adequate
- ◯ I don't know
- ◯ N/A

9. How often does your clinic practice partner with external community programs focused on helping people experiencing loneliness or social isolation?

- Never
- Rarely
- Sometimes
- Often
- Always
- I don't know
- N/A

10. How often do you teach about or discuss loneliness and social isolation with your family medicine students and/or residents?

- Never
- Rarely
- Sometimes
- Often
- Always
- Does not apply - I am not directly teaching/ precepting
